# DNA methylation mediates the effect of exposure to prenatal maternal stress on cytokine production in children at age 13½ years: Project Ice Storm

**DOI:** 10.1186/s13148-016-0219-0

**Published:** 2016-05-12

**Authors:** Lei Cao-Lei, Franz Veru, Guillaume Elgbeili, Moshe Szyf, David P. Laplante, Suzanne King

**Affiliations:** Department of Psychiatry, McGill University and Douglas Hospital Research Centre, 6875 LaSalle Blvd, Montreal, Quebec H4H 1R3 Canada; Douglas Hospital Research Centre, Montreal, Quebec Canada; Department of Pharmacology and Therapeutics and Sackler Program for Epigenetics and Developmental Psychobiology, McGill University, Montreal, Quebec Canada

**Keywords:** Prenatal maternal stress, DNA methylation, Mediation effect, Cytokines, NF-κB signaling, IFN-γ

## Abstract

**Background:**

Prenatal maternal stress (PNMS) is an important programming factor of postnatal immunity. We tested here the hypothesis that DNA methylation of genes in the NF-κB signaling pathway in T cells mediates the effect of objective PNMS on Th1 and Th2 cytokine production in blood from 13½ year olds who were exposed in utero to the 1998 Quebec ice storm.

**Results:**

Bootstrapping analyses were performed with 47 CpGs across a selection of 20 genes for Th1-type cytokines (IFN-γ and IL-2) and Th2-type cytokines (IL-4 and IL-13). Six CpGs in six different NF-κB signaling genes (*PIK3CD*, *PIK3R2*, *NFKBIA*, *TRAF5*, *TNFRSF1B*, and *LTBR*) remained as significant negative mediators of objective PNMS on IFN-γ secretion after correcting for multiple comparisons. However, no mediation effects on IL-2, IL-4 and IL-13 survived Bonferroni correction.

**Conclusions:**

The present study provides preliminary evidence supporting the mediating role of DNA methylation in the association between objective aspects of PNMS and child immune states, favoring a Th2 shift.

**Electronic supplementary material:**

The online version of this article (doi:10.1186/s13148-016-0219-0) contains supplementary material, which is available to authorized users.

## Background

The prenatal programming of postnatal disease hypothesis [1] postulates that many physical conditions, including immune disorders, have a prenatal etiological component. Prenatal maternal stress (PNMS) has proven to be an important programming factor of postnatal immunity, as shown in the studies of the development of immune-related disorders in postnatal life such as asthma [2–7]. Factors that need to be considered regarding the effects of stress on immunity are as follows: (1) The induction, progression, and homeostatic regulation of the immune response depend on signaling mechanisms. (2) Immune responses depend on the “chief” cells of the immune system: T helper (Th) lymphocytes. Th cells differentiate into two main types of effector cells depending on the type of immune challenge: Th1 differentiation evolved to control intracellular invaders, while Th2 developed to face extracellular parasites. (3) Intercellular signaling is carried out through cytokine production. (4) Chronic stress in adults elicits a shift in the balance between Th1-derived cytokines and Th2-derived cytokines.

However, one cannot extrapolate the effects of stress on the fully developed adult immune system to the effects of maternal stress on the unborn child, due to the physiological changes occurring in stress physiology during pregnancy [8], the dynamic shielding from the maternal environment that the placenta provides to the fetus [9], and the developmental stage [10] of the immune system at the time of exposure to PNMS. Despite these considerations, PNMS has demonstrated a pervasive influence on immunity per se, altering a broad array of functions at the molecular and cellular levels. We previously reviewed the literature on the influence of PNMS on immunity in laboratory animals [11] where these changes have been documented more systematically than in humans. Using a framework that standardized prenatal timing across common developmental windows of vulnerability, we integrated findings from studies using five different species of mammals [11]. In brief, the immunological consequences of PNMS exposure can be observed in the offspring’s cytokine production, lymphocyte population proportions, mitogen-induced proliferative responses, antibody production, cell-mediated responses, natural killer cell cytotoxicity, and macrophage activity. The most consistently replicated findings have been observed in cytokine homeostasis, where PNMS induces a Th2 cytokine shift (adaptive immunity) and excessive pro-inflammatory cytokine responses (innate immunity).

Although limited in number, results from human studies are consistent with animal findings. Adult women who were exposed to PNMS (negative life events) demonstrated a Th2 cytokine shift when compared to controls [12]. Likewise, in a cohort of pregnant women exposed to multiple stressors, cytokine production in cord blood stimulated by lipopolysaccharide (LPS) evidenced an enhanced pro-inflammatory state, while levels after phytohemagglutinin (PHA) stimulation suggested an immune imbalance favoring Th2 responses [13]. However, these studies lacked random assignment as done in animal studies and precise dating of the stressor exposure in pregnancy.

Project Ice Storm, a prospective longitudinal study, serves as a human stress model which is able to address these issues. Natural disasters can act like natural experiments and provide the opportunity to study the effects of PNMS from an event whose genesis is totally independent of the parents’ influence and whose degree of hardship is often quasi-randomly distributed in the population. By using a natural disaster as our stressor, we were able to separate the pregnant women’s stress experience into components, such as objective levels of hardship and subjective distress reactions to those hardships. Evaluation of objective stress involves examining the specific events experienced by the pregnant women during the crisis, such as duration of power loss, loss of income, and amount of change to daily routines. The second component, subjective stress, involves assessing the pregnant women’s psychological response to the event. Being uncorrelated with socioeconomic status or maternal traits, we consider our measure of objective hardship from the ice storm to be a quasi-randomly assigned and independent stressor.

Previously, we reported that PNMS from the ice storm predicted significant alterations in lymphocytes and cytokine production at the innate and adaptive arms of immunity [14]. Specifically, the greater the objective degree of exposure to the ice storm (objective PNMS) experienced by the pregnant mother, the lower the total and CD4+ lymphocyte proportions, the higher the TNF-α, IL-1β, and IL-6 levels, and the greater the enhancement of the Th2 cytokines IL-4 and IL-13 in the child aged 13½ years; maternal subjective distress was not significantly associated with cytokine levels. These results show that the objective degree of hardship resulting from this natural disaster was significant as a programming factor that can produce long-lasting consequences for immunity, potentially explaining non-genetic variability in immune-related disorders. These immunological alterations have significant clinical implications, considering the central role that enhanced Th2 responses have in the pathophysiology of allergic disorders in general [15], and asthma in particular [16]. Moreover, pro-inflammatory cytokine changes have been documented in some subtypes of asthma [17].

However, the mechanisms underlying the association between PNMS and alteration of immune function still remain unclear. One of the most documented putative mechanisms is DNA methylation. A rich literature documents epigenetic regulation of immune function and immune-related diseases. For example, epigenetic mechanisms have been involved in regulation of IFN-γ expression in CD8 T cells (reviewed in [18]). A study reporting the methylcytosine dioxygenase Tet2-promoted DNA demethylation and activation of cytokine gene expression in T cells [19] provides a global view of a novel epigenetic modification that occurs during Th cell differentiation, unveiling a functional role for Tet2-mediated active DNA demethylation in the function of Th cells both in vitro and in vivo. Furthermore, CD4+ T cells from patients with juvenile idiopathic arthritis (JIA), the most common autoimmune rheumatic disease of childhood, showed reduction of IL-32 through DNA methylation [20]. Likewise, the reduced DNA methylation level of the IL-32 gene was also observed in another study of JIA [21], focusing on epigenetic modification in genome-wide DNA methylation levels. Moreover, as reviewed by Kabesch et al. [22], multiple studies have demonstrated a well-established relationship between epigenetic mechanisms and asthma in children. Together, as demonstrated herein, epigenetic variation, particularly DNA methylation, plays a crucial role in immunity.

Although associations between epigenetic profiles and health outcomes have been established (as discussed above), what is rarely demonstrated is the extent to which DNA methylation mediates the effects of a well-documented prenatal environmental event on a postnatal health outcome in human offspring. Natural disasters offer a unique opportunity to examine relationships between exposures, changes in DNA methylation, and the phenotypic outcomes in a quasi-experimental design. Project Ice Storm pilot data show that the objective degree of maternal exposure to the ice storm results in significant changes in the offspring’s epigenetic signatures in T cells at 13½ years of age [23] in genes predominantly associated with immune function and, to a lesser extent, metabolism; there were no significant associations between subjective PNMS and DNA methylation. Most recently, we have reported that specific DNA methylation signatures mediate the effects of objective exposure on these children’s body composition as reflected in body mass index (BMI) and central adiposity [24]. Whether, and to what extent, DNA methylation levels in T cells can mediate the effect of PNMS on cytokine production (i.e., Th1-Th2 balance) remains to be investigated.

The goal of the present study was to determine the extent to which alterations in DNA methylation in immune-regulating genes (i.e., within the NF-κB signaling pathway) may mediate the effects of objective PNMS experienced by pregnant women during the 1998 ice storm on the production of Th1-type cytokines (IFN-γ and IL-2) and Th2-type cytokines (IL-4 and IL-13) in their children at age 13½. Given that we have not found that maternal subjective distress from the ice storm predicted either DNA methylation [23] or cytokine secretion [14], there was no reason to test mediation effects for subjective PNMS.

## Methods

### Participants

Participants were recruited from the longitudinal Project Ice Storm [25] which focuses on disaster-related PNMS and child development. The mothers in the study had been in their first, second, or third trimester of pregnancy on January 9, 1998 (the peak of the ice storm). We also included mothers who conceived within 3 months of the storm because preconception exposure to stress has been reported to have cross-generational effects in both animal and human studies [26–30]. A subsample of 37 children were involved in the immune study [14], and among these children, 34 DNA samples were subjected to genome-wide DNA methylation analysis [23]. In the current study, as one child’s cytokine data were removed because the values were extremely high, the DNA methylation data of 33 children were used for further analyses. The key characteristics of the 33 participating children and their mothers, such as objective PNMS, birth weight, and socioeconomic status, did not differ significantly from those of the rest of the Project Ice Storm cohort. All phases of this study were approved by the Research Ethics Board of the Douglas Hospital Research Centre (REB#: 11/33). We obtained informed written consent from parents at every assessment and informed written assent from the children at the age 13½ assessment.

### Instruments and measures

#### PNMS variables

The degree of PNMS, as well as family demographic variables, was assessed in a postal questionnaire mailed to participants on June 1, 1998. Objective PNMS was calculated using the mothers’ responses to questionnaire items tapping into categories of exposure used in other disaster studies: threat, loss, scope, and change [31]. Because each natural disaster presents unique experiences to the exposed population, questions pertaining to each of the four categories must be tailor-made. Each of the four dimensions was scored on a scale of 0–8, ranging from low exposure to high exposure. A total objective hardship score (Storm32) was computed by summing scores from all four dimensions using McFarlane’s approach [32]. Details of the Storm32 items and scoring are presented elsewhere [33].

Subjective PNMS was assessed using the validated French version [34] of the widely used Impact of Event Scale-Revised (IES-R) [35]. The 22-item scale describes symptoms from three categories relevant to post-traumatic stress disorder: intrusive thoughts, hyperarousal, and avoidance. The IES-R instructions for respondents allow investigators or clinicians to “write in” the traumatic event in question. Participants responded on a five-point Likert scale, from “Not at all” to “Extremely”, the extent to which each item described how they felt over the preceding seven days in response to the ice storm crisis. We used the total score in all analyses.

#### Maternal variables

The level of maternal psychological functioning was assessed with a validated French version of the widely used General Health Questionnaire-28 (GHQ) [36]. The GHQ is a self-report screening tool for psychiatric symptoms and includes seven items in each of the depression, anxiety, dysfunction, and somatization sub-scales. Items are scored on a four-point Likert scale indicating the degree to which each symptom was experienced in the preceding 2 weeks. In the present study, each item was re-scored as either 0 (a rating of 0 or 1) or 1 (a rating of 2 or 3), according to the Goldberg method [36], resulting in a minimum possible score of 0 and a maximum possible score of 28. The total score was used in analyses. The GHQ was included in the June 1998 questionnaire and also when their children were 13½ years of age.

Data on maternal and paternal age and education, and parental job classification, were collected in June 1998. The Hollingshead Socioeconomic Index (SES) was computed using these data [37].

The number of obstetrical complications, including flu with fever, was determined by maternal recall using an adaptation of the scale used by Kinney [38] in our 6-month postpartum questionnaire and verified using hospital records. We used the total number of obstetrical complications experienced by the women that were rated as moderate-to-severe using the McNeil-Sjöström Scale for Obstetrical Complications [39].

The children’s birth weight, birth length, and gestational age were obtained from maternal reports (transcribed from Quebec birth records given at discharge) in the 6-month postpartum questionnaire and from hospital records. Birth ponderal index was also calculated (100×(birth weight (g)/birth length^3^ (cm)).

### Blood samples and T cell isolation at 13½ years

Blood was collected from the subjects for T cell isolation and DNA extraction using methods which have been described previously [23]. Briefly, T cells were isolated from peripheral blood mononuclear cells (PBMCs) by immunomagnetic separation with Dynabeads CD3 (Dynal, Invitrogen). DNA extraction from T cells was performed using Wizard Genomic DNA Purification kit (Promega) according to the manufacturer’s instructions.

### Cytokine production measures of 13½ year-old children

Cytokine levels were measured using bead-based immunoassays as previously described [14]. Briefly, cytokines were obtained from whole-blood cultures stimulated with PHA, which promotes adaptive immune responses (and thus, Th1 and Th2 cytokine secretion). Cytokine levels were then measured from culture supernatants using bead-based immunoassays (CBA: Cytometric Bead Array Flex-sets, BD Biosciences, San Diego, CA) following the manufacturer’s protocol. Since DNA methylation levels were obtained from T cells, we focused on exploring the potential mediation effect of DNA methylation on Th1- and Th2-type cytokine secretion. The Th1-type cytokine detecting panel was comprised of IFN-γ and IL-2; Th2-type panel was comprised of IL-4 and IL-13. Therefore, four cytokine levels were used for the mediation analyses.

### Selection of candidate genes for testing mediation by DNA methylation

**Fig. 1:**
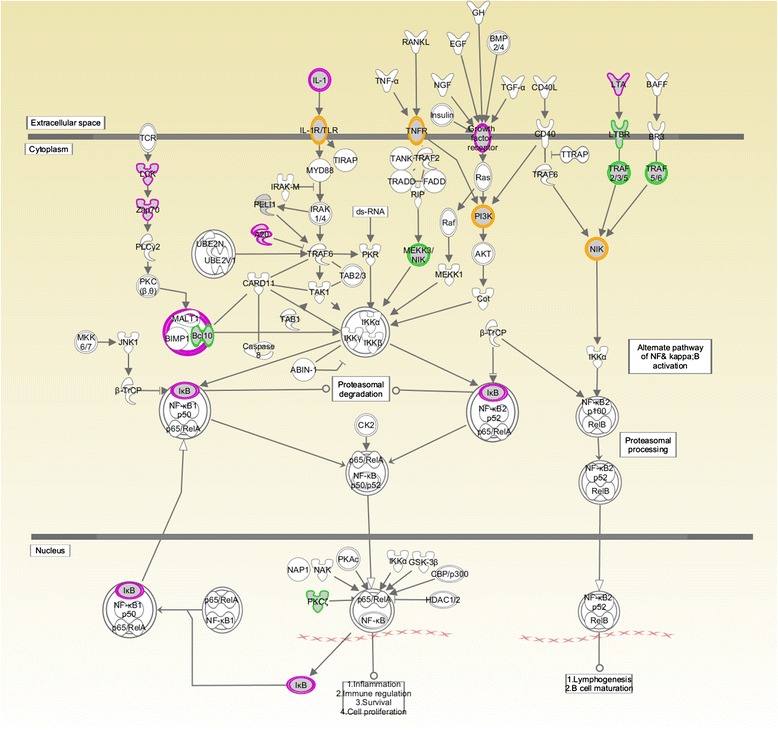
NF-κB signaling pathway with genes associated with objective PNMS. Among the 20 genes selected from NF-κB signaling pathway, *DDR1*, *FLT1*, *IGF1R*, *IL1R2*, *LCK*, *LTA*, *MAP4K4*, *NFKBIA*, *PIK3CD*, *TGFBR2*, *TNFAIP3*, and *ZAP70* were greater methylated; *BCL10*, *LTBR*, *MAP3K14*, *PIK3R2*, *PRKCZ*, *TLR6* and *TRAF5* were lesser methylated; *TNFRSF1B* was either greater or lesser methylated depending on the different CpG sites. The gene family *A20* (*TNFAIP3*), growth factor receptor (*DDR1*, *FLT1*, *IGF1R*, and *TGFBR2*), *IkB* (*NFKBIA*), *LCK*, *LTA*, and *ZAP70* are highlighted in red. The gene family *BCL10*, *LTBR*, *MEKK3/NIK* (*MAP3K14*), *PRKCZ*, *TRAF2/3/5*, and *TRAF5/6* are highlighted in green. The gene family *IL-1R/TLR* (*IL1R2* and *TLR6*), *NIK* (*MAP3K14* and *MAP4K4*), *P13K* (*PIK3CD* and *PIK3R2*), and *TNFR* (*TNFRSF1B*) are highlighted in orange.

In order to determine the extent to which gene methylation mediates the association between objective PNMS and cytokine production at 13½ years, we tested genes that we have previously shown to have T cell methylation signatures that are correlated with objective PNMS levels in this sample (see [23] for the detailed description). We then matched the genes whose methylation had been significantly correlated with objective PNMS to the NF-κB signaling pathway as classified by IPA software (www.ingenuity.com), given the important role of NF-κB, a major transcription factor that regulates genes responsible for both the innate and adaptive immune responses. In total, 20 genes (47 CpGs) were selected. Although we found several polymorphic CpGs among the 47 CpGs, these CpGs were still used in the mediation analyses because of their very low alternative allele frequencies which would not be expected to have a major effect on methylation levels [40]. The corresponding genes of these CpGs and functions of these genes are listed in Additional file 1. The NF-κB pathway, with the 20 genes associated with objective PNMS, is shown in Fig. 1.

### Statistical analysis

We used bootstrap methods, a routinely used approach, to determine the significance of mediation effects [41]. Bootstrapping involves randomly resampling with replacement from the dataset to compute the desired statistic in each resample, providing confidence intervals by which the significance of a mediation effect can be determined. We tested whether objective PNMS had an effect on cytokine production through each CpG site associated with NF-κB signaling pathway (47 CpG sites across 20 genes). To that end, 95 % bias-corrected bootstrap confidence intervals were computed, as explained by Hayes [42]. The PROCESS procedure for SPSS [42] was used to conduct the analyses. Each bootstrap procedure resampled the initial sample 10,000 times. In order to be able to recover the same results, the random seed for all bootstraps was fixed at 1,509,805,407, an integer randomly generated between 1 and 2,000,000,000, prior to the first bootstrap. A mediation effect was considered significant if 0 was not included in the bootstrap confidence interval.

To adjust significance criteria as a function of the number of analyses conducted for each cytokine outcome, the confidence interval ranges were corrected using Bonferroni. However, because two separate simple mediation analyses including two highly correlated mediators most likely lead to very similar results, traditional Bonferroni correction would be too conservative an approach. Thus, we used the method proposed by Li and Ji [43] to calculate the effective number (M_eff_) of independent tests, essentially reducing the denominator to a number that more accurately reflects the number of intercorrelated groups of CpG mediators. This method has been applied to microarray data using Pearson correlations [43]. In order to apply this method to the current study, four steps were performed. The first step was to calculate the correlations among the methylation levels of the 47 CpGs used in the mediation analyses; most of the 47 CpG site methylation levels were highly correlated with each other (*|p|* values: min. = 0.33; max. = 0.98; median = 0.81). The second step was to compute the 47 eigenvalues from that correlation matrix. The third step was then to modify each eigenvalue following Li and Ji’s formula: $f(x)=I\left(x\ge 1\right)+\left(x-\left\lfloor x\right\rfloor \right)$. Basically, the new value’s integer portion is set to 0 if the eigenvalue was smaller than 1 and is set to 1 otherwise while the decimals remain unchanged. The last step consisted in summing the 47 new values to compute the effective number of independent tests (*M*_eff_) and use that *M*_eff_ as the correction factor in the Bonferroni method for multiple testing corrections. The resulting *M*_eff_ was 10. Therefore, the adjusted confidence intervals were recomputed for 10 tests at a 99.5 % level using Bonferroni.

To take into account the different variability in methylation across CpG sites, standardized mediation effects were also computed. Standardized mediation effects are computed by standardizing the variables included in the model prior to running the analyses. All the other effects presented in the mediation results section are unstandardized. All analyses were completed with SPSS 20.0 (SPSS, Chicago, IL).

## Results

### Participants’ characteristics

At the time of assessment, the 33 subjects were, on average, 13.3 years of age (SD = 0.3). There were 18 boys and 15 girls. The mothers of the subjects had been in their first (*n* = 11), second (*n* = 9), or third (*n* = 7) trimester of pregnancy on January 9, 1998 or conceived within 3 months of the storm (*n* = 6). Descriptive statistics for the participants are shown in Table 1. The mothers experienced a range of severity of objective hardship. They also experienced a range of subjective distress as assessed by the IES-R; five of the mothers obtained a score at or above 22 which is the screening cut-off for potential PTSD.

**Table 1:** The characteristics of the mothers and children

	Minimum	Maximum	Mean	Standard deviation
Prenatal maternal stress (PNMS)				
Objective hardship (Storm32)	5.0	21.0	11.5	4.1
Subjective distress (IES-R)	0.0	40.0	10.1	9.4
Mothers				
Socioeconomic status	11.0	65.0	27.6	11.1
Obstetrical complications	0.0	12.0	4.7	3.1
Children’s birth characteristics				
Birth weight (g)	1655.0	4432.0	3426.3	649.4
Birth length (cm)	41.0	56.5	50.5	3.1
Birth ponderal index	20.2	34.5	26.7	3.6
Children at age 13½				
IFN-γ	11.9	119.7	32.6	21.2
IL-2	29.4	266.5	96.6	51.8
IL-4	3.1	24.5	7.4	4.3
IL-13	13.9	64.7	27.6	10.4

### Correlation analyses

Correlations between objective PNMS and Th2 and Th1 adaptive cytokine production variables were analyzed. Objective PNMS was significantly positively associated with both of the Th2 cytokines: IL-4 (*r* = 0.365, *p* = 0.037) and IL-13 (*r* = 0.345, *p* = 0.049). Neither of the Th1 adaptive cytokine levels was significantly associated with objective PNMS: IFN-γ (*r* = 0.207, *p* = 0.247) and IL-2 (*r* = 0.284, *p* = 0.110). Furthermore, objective PNMS was not associated with subjective distress (IES-R) (*r* = 0.164, *p* = 0.361), socioeconomic status (SES) (*r* = 0.269, *p* = 0.130), nor with obstetrical complications (*r* = 0.269, *p* = 0.130).

### Mediation analyses

**Fig. 2:**
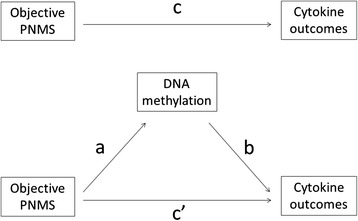
Mediation analysis on the relationship between exposure to objective PNMS and cytokine production. Path “*a*” is the effect of the objective PNMS (predictor variable) on the DNA methylation (mediator), path “*b*” is the effect of the DNA methylation on cytokine production (outcome variable) controlling for the objective PNMS, and path “*c’* ” is the direct effect of the objective PNMS on cytokine production controlling for the DNA methylation. The path “a*b” indicates the mediating effect of objective PNMS on cytokine production through DNA methylation (mediator). Path “*c*” is the total effect of objective PNMS on cytokine production.

The theoretical model of the mediation analysis is presented in Fig. 2. We performed bootstrapping analyses with the 47 CpGs across the selected 20 genes from the NF-κB signaling pathway to test whether the associations between objective PNMS and Th1- and Th2-type cytokine production (IFN-γ, IL-2, IL-4, and IL-13), as reported in our previous study [14] are mediated through DNA methylation.

Tables 2, 3, 4, and 5 present the results for each of the four cytokines. Each table presents the effects of objective PNMS on DNA methylation (Fig. 2, path *a*), the effect of DNA methylation on cytokine production controlling for objective PNMS (path *b*), the direct effect of PNMS on the cytokine production controlling for DNA methylation (path *c’*), the mediation effect (path a*b), and the standardized mediation effect through each CpG site. The tables present the results that were significant initially; results that were significant after Bonferroni correction for multiple testing are highlighted in italics.

**Table 2:** Significant mediating effects of gene methylation on IFN-γ outcome

Gene	CpG site	DNA methylation				IFN-γ outcome							Mediation effect				
		*R* ^2^	Storm32			*R* ^2^	DNA methylation			Storm32							
			Effect (*a*)	SE	*p* val.		Effect (*b*)	SE	*p* val.	Effect (*c'*)	SE	*p* val.	Effect (a*b)	Boot SE	Boot LLCI	Boot ULCI	Standardized effect
DDR1	cg11975790	0.1813	0.0527	0.0201	0.0135	0.1749	−0.4065	0.1856	0.0364	0.0474	0.0230	0.0478	−0.0214	0.0116	−0.0537	−0.0045	−0.170991
FLT1	cg24850711	0.2527	0.0606	0.0187	0.0029	0.1142	−0.3210	0.2065	0.1306	0.0454	0.0249	0.0782	−0.0195	0.0129	−0.0540	−0.0010	−0.155283
IGF1R	cg13297560	0.1322	0.0388	0.0178	0.0375	0.1376	−0.3883	0.2139	0.0795	0.0410	0.0228	0.0822	−0.0151	0.0113	−0.0475	−0.0007	−0.120139
IL1R2	cg12910851	0.1628	0.0353	0.0144	0.0199	0.2001	−0.6216	0.2560	0.0214	0.0479	0.0224	0.0406	−0.0219	0.0151	−0.0659	−0.0031	−0.174821
LCK	cg17078393	0.2561	0.0659	0.0202	0.0027	0.1783	−0.4104	0.1846	0.0339	0.0530	0.0241	0.0353	−0.0271	0.0139	−0.0640	−0.0055	−0.21591
	cg05350315	0.1949	0.0711	0.0260	0.0101	0.1651	−0.3033	0.1447	0.0447	0.0475	0.0233	0.0503	−0.0216	0.0145	−0.0629	−0.0019	−0.172012
	cg11683242	0.1681	0.0443	0.0177	0.0178	0.2545	−0.5858	0.2007	0.0066	0.0519	0.0217	0.0231	−0.0259	0.0126	−0.0599	−0.0073	−0.206796
	cg14781242	0.2081	0.0505	0.0177	0.0076	0.1625	−0.4395	0.2124	0.0472	0.0482	0.0235	0.0495	−0.0222	0.0109	−0.0519	−0.0063	−0.177247
LTA	cg16280132	0.3331	0.0652	0.0166	0.0004	0.1463	−0.4366	0.2291	0.0663	0.0544	0.0259	0.0440	−0.0285	0.0152	−0.0654	−0.0047	−0.227195
	cg09736959	0.2952	0.0758	0.0210	0.0011	0.1433	−0.3391	0.1809	0.0706	0.0517	0.0252	0.0494	−0.0257	0.0150	−0.0628	−0.0014	−0.20498
	cg24216966	0.2611	0.0886	0.0268	0.0024	0.1401	−0.2620	0.1423	0.0755	0.0492	0.0247	0.0555	−0.0232	0.0144	−0.0628	−0.0022	−0.185282
	cg01157951	0.2634	0.0663	0.0199	0.0023	0.1334	−0.3402	0.1922	0.0869	0.0485	0.0248	0.0600	−0.0225	0.0140	−0.0592	−0.0010	−0.179876
	cg21999229	0.2593	0.1120	0.0340	0.0025	0.1287	−0.1940	0.1129	0.0961	0.0477	0.0248	0.0643	−0.0217	0.0142	−0.0592	−0.0002	−0.17327
	cg14597739	0.2618	0.1198	0.0361	0.0023	0.1504	−0.2043	0.1049	0.0608	0.0504	0.0246	0.0487	−0.0245	0.0156	−0.0674	−0.0021	−0.195257
	cg16219283	0.2575	0.1038	0.0316	0.0026	0.1430	−0.2250	0.1202	0.0710	0.0493	0.0246	0.0540	−0.0234	0.0151	−0.0641	−0.0010	−0.186303
	cg13815684	0.2571	0.0877	0.0268	0.0026	0.1569	−0.2838	0.1410	0.0531	0.0509	0.0244	0.0456	−0.0249	0.0141	−0.0635	−0.0038	−0.198557
	cg10476003	0.2229	0.0794	0.0266	0.0055	0.1749	−0.3071	0.1402	0.0364	0.0504	0.0236	0.0410	−0.0244	0.0138	−0.0656	−0.0048	−0.194562
	cg09621572	0.2220	0.1022	0.0343	0.0056	0.1447	−0.2091	0.1107	0.0686	0.0473	0.0240	0.0578	−0.0214	0.0141	−0.0609	−0.0024	−0.170417
	cg17169196	0.2233	0.0894	0.0299	0.0055	0.1638	−0.2615	0.1255	0.0459	0.0493	0.0237	0.0464	−0.0234	0.0138	−0.0624	−0.0042	−0.186458
	cg02402436	0.2261	0.0932	0.0310	0.0052	0.1323	−0.2173	0.1236	0.0890	0.0462	0.0242	0.0661	−0.0203	0.0129	−0.0574	−0.0018	−0.161559
	cg14441276	0.2038	0.0741	0.0263	0.0084	0.1409	−0.2678	0.1448	0.0742	0.0458	0.0238	0.0634	−0.0199	0.0136	−0.0591	−0.0010	−0.158425
	cg11586857	0.1575	0.0561	0.0233	0.0222	0.1857	−0.3647	0.1590	0.0290	0.0464	0.0225	0.0477	−0.0205	0.0123	−0.0566	−0.0033	−0.163339
	cg14437551	0.1758	0.0986	0.0383	0.0151	0.1758	−0.2304	0.0961	0.0230	0.0487	0.0226	0.0393	−0.0227	0.0140	−0.0635	−0.0035	−0.181167
*LTBR*	*cg23079808*	*0.1487*	*−0.0474*	*0.0204*	*0.0267*	*0.2281*	*0.4754*	*0.1772*	*0.0118*	*0.0485*	*0.0218*	*0.0337*	*−0.0225*	*0.0123*	*−0.0521*	*−0.0046*	*−0.179865*
MAP3K14	cg16826777	0.1665	−0.0516	0.0207	0.0184	0.2136	0.4488	0.1759	0.0161	0.0491	0.0222	0.0349	−0.0231	0.0123	−0.0568	−0.0055	−0.184658
NFKBIA	*cg00689225*	*0.2086*	*0.0668*	*0.0234*	*0.0075*	*0.2304*	*−0.4173*	*0.1544*	*0.0112*	*0.0538*	*0.0226*	*0.0236*	*−0.0279*	*0.0127*	*−0.0630*	*−0.0095*	*−0.222303*
	cg16518861	0.1288	0.0439	0.0205	0.0403	0.2475	−0.4968	0.1740	0.0077	0.0478	0.0213	0.0322	−0.0218	0.0127	−0.0579	−0.0046	−0.173912
PIK3CD	*cg01320698*	*0.3012*	*0.0487*	*0.0133*	*0.0009*	*0.2948*	*−0.8481*	*0.2591*	*0.0027*	*0.0673*	*0.0230*	*0.0065*	*−0.0413*	*0.0172*	*−0.0857*	*−0.0155*	*−0.329546*
	cg07499142	0.1747	0.0599	0.0234	0.0155	0.1688	−0.3416	0.1603	0.0413	0.0464	0.0230	0.0522	−0.0205	0.0109	−0.0480	−0.0034	−0.163265
PIK3R2	*cg11953794*	*0.2101*	*−0.0753*	*0.0262*	*0.0073*	*0.2804*	*0.4185*	*0.1330*	*0.0037*	*0.0575*	*0.0218*	*0.0133*	*−0.0315*	*0.0134*	*−0.0659*	*−0.0109*	*−0.251347*
	cg04610450	0.1700	−0.0442	0.0176	0.0171	0.2369	0.5647	0.2045	0.0097	0.0510	0.0219	0.0272	−0.0250	0.0127	−0.0600	−0.0068	−0.199292
PRKCZ	cg02481000	0.1183	−0.0434	0.0213	0.0501	0.1988	0.4175	0.1728	0.0220	0.0441	0.0218	0.0523	−0.0181	0.0120	−0.0530	−0.0024	−0.14457
TGFBR2	cg04916416	0.1641	0.0412	0.0167	0.0193	0.1691	−0.4793	0.2246	0.0411	0.0457	0.0228	0.0543	−0.0197	0.0114	−0.0514	−0.0032	−0.157375
TLR6	cg14578677	0.1978	−0.0707	0.0256	0.0095	0.1637	0.3060	0.1470	0.0460	0.0476	0.0234	0.0506	−0.0216	0.0130	−0.0563	−0.0029	−0.172535
TNFAIP3	cg22014112	0.1467	0.0563	0.0244	0.0278	0.1868	−0.3499	0.1519	0.0284	0.0457	0.0223	0.0497	−0.0197	0.0131	−0.0576	−0.0024	−0.157265
TNFRSF1B	cg05599723	0.1221	−0.0465	0.0224	0.0462	0.1850	0.3790	0.1657	0.0294	0.0436	0.0221	0.0573	−0.0176	0.0111	−0.0496	−0.0024	−0.140605
	cg15526535	0.1349	0.0389	0.0177	0.0355	0.1947	−0.4959	0.2086	0.0240	0.0453	0.0221	0.0493	−0.0193	0.0113	−0.0496	−0.0038	−0.153835
	*cg22677556*	*0.2258*	*0.0679*	*0.0226*	*0.0052*	*0.1711*	*−0.3567*	*0.1656*	*0.0394*	*0.0502*	*0.0237*	*0.0424*	*−0.0242*	*0.0107*	*−0.0539*	*−0.0084*	*−0.193363*
TRAF5	*cg10721755*	*0.1654*	*−0.0516*	*0.0208*	*0.0188*	*0.2716*	*0.5175*	*0.1686*	*0.0045*	*0.0527*	*0.0214*	*0.0198*	*−0.0267*	*0.0140*	*−0.0644*	*−0.0070*	*−0.212858*
ZAP70	cg08859278	0.1844	0.0475	0.0179	0.0126	0.2533	−0.5756	0.1979	0.0068	0.0533	0.0219	0.0211	−0.0273	0.0135	−0.0627	−0.0073	−0.2181

Mediation effects that survived Bonferroni correction are presented in italics

*BCL10* B cell CLL/lymphoma 10, *DDR1* discoidin domain receptor tyrosine kinase 1, *FLT1* Fms-related tyrosine kinase 1, *IGF1R* insulin-like growth factor 1 receptor, *IL1R2* interleukin 1 receptor, type II, *LCK* lymphocyte-specific protein tyrosine kinase, *LTA* lymphotoxin alpha, *LTBR* lymphotoxin beta receptor (TNFR superfamily, member 3), *MAP3K14* mitogen-activated protein kinase kinase kinase 14, *MAP4K4* mitogen-activated protein kinase kinase kinase kinase 4, *NFKBIA* nuclear factor of kappa light polypeptide gene enhancer in B cell inhibitor, alpha, *PIK3CD* phosphatidylinositol-4,5-bisphosphate 3-kinase, catalytic subunit delta, *PIK3R2* phosphoinositide-3-kinase, regulatory subunit 2 (beta), *PRKCZ* protein kinase C, zeta, *TGFBR2* transforming growth factor, beta receptor II, *TLR6* Toll-like receptor 6, *TNFAIP3* tumor necrosis factor, alpha-induced protein 3, *TNFRSF1B* tumor necrosis factor receptor superfamily, member 1B, *TRAF5* TNF receptor-associated factor 5, *ZAP70* zeta-chain (TCR)-associated protein kinase 70 kDa, *SE* standard error, *LLCI* lower limit confidence interval, *ULCI* upper limit confidence interval

**Table 3:** Significant mediating effects of gene methylation on IL-2 outcome

Gene	CpG site	DNA methylation				IL-2 outcome							Mediation effect				
		*R* ^2^	Storm32			*R* ^2^	DNA methylation			Storm32							
			Effect (*a*)	SE	*p* val.		Effect (*b*)	SE	*p* val.	Effect (*c'*)	SE	*p* val.	Effect (a*b)	Boot SE	Boot LLCI	Boot ULCI	Standardized Effect
DDR1	cg11975790	0.1813	0.0527	0.0201	0.0135	0.1458	0.2919	0.1926	0.1402	0.0209	0.0238	0.3882	0.0154	0.0109	0.0001	0.0449	0.12033
LCK	cg05350315	0.1949	0.0711	0.0260	0.0101	0.1468	0.2279	0.1492	0.1372	0.0201	0.0240	0.4104	0.0162	0.0123	0.0001	0.0504	0.126718
LTA	cg21999229	0.2593	0.1120	0.0340	0.0025	0.1579	0.1881	0.1132	0.1071	0.0152	0.0249	0.5459	0.0211	0.0131	0.0015	0.0559	0.164705
	cg14597739	0.2618	0.1198	0.0361	0.0023	0.2009	0.2206	0.1037	0.0418	0.0098	0.0243	0.6884	0.0264	0.0145	0.0061	0.0681	0.206707
	cg16219283	0.2575	0.1038	0.0316	0.0026	0.1693	0.2163	0.1208	0.0833	0.0138	0.0247	0.5799	0.0224	0.0142	0.0020	0.0620	0.175555
	cg17169196	0.2233	0.0894	0.0299	0.0055	0.1498	0.2020	0.1292	0.1284	0.0182	0.0244	0.4616	0.0181	0.0134	0.0000	0.0566	0.141177
	cg02402436	0.2261	0.0932	0.0310	0.0052	0.1701	0.2221	0.1234	0.0818	0.0156	0.0242	0.5244	0.0207	0.0113	0.0038	0.0528	0.161848
	cg14441276	0.2038	0.0741	0.0263	0.0084	0.1522	0.2338	0.1467	0.1216	0.0189	0.0241	0.4380	0.0173	0.0119	0.0004	0.0513	0.135532
TGFBR2	cg04916416	0.1641	0.0412	0.0167	0.0193	0.1289	0.3029	0.2346	0.2065	0.0238	0.0238	0.3259	0.0125	0.0090	0.0006	0.0370	0.097494

None of the mediation effects survived Bonferroni correction

**Table 4:** Significant mediating effects of gene methylation on IL-4 outcome

Gene	CpG site	DNA methylation				IL-4 outcome							Mediation effect				
		*R* ^2^	Storm32			*R* ^2^	DNA methylation			Storm32							
			Effect (*a*)	SE	*p* val.		Effect (*b*)	SE	*p* val.	Effect (*c'*)	SE	*p* val.	Effect (a*b)	Boot SE	Boot LLCI	Boot ULCI	Standardized effect
BCL10	cg01636910	0.1332	−0.0506	0.0232	0.0367	0.2035	−0.2347	0.1441	0.1139	0.0297	0.0200	0.1466	0.0119	0.0091	0.0006	0.0368	0.104041
IGF1R	cg13297560	0.1322	0.0388	0.0178	0.0375	0.2072	0.3125	0.1866	0.1044	0.0295	0.0199	0.1489	0.0121	0.0093	0.0004	0.0386	0.106277
LCK	cg17078393	0.2561	0.0659	0.0202	0.0027	0.2394	0.3308	0.1616	0.0495	0.0198	0.0211	0.3547	0.0218	0.0132	0.0037	0.0563	0.191284
	cg05350315	0.1949	0.0711	0.0260	0.0101	0.2457	0.2648	0.1252	0.0428	0.0228	0.0202	0.2673	0.0188	0.0119	0.0029	0.0524	0.165089
MAP3K14	cg16826777	0.1665	−0.0516	0.0207	0.0184	0.2167	−0.2857	0.1597	0.0838	0.0269	0.0202	0.1931	0.0147	0.0093	0.0018	0.0396	0.129202
PIK3CD	cg07499142	0.1747	0.0599	0.0234	0.0155	0.2021	0.2300	0.1429	0.1178	0.0278	0.0205	0.1842	0.0138	0.0090	0.0003	0.0373	0.120821
PIK3R2	cg11953794	0.2101	−0.0753	0.0262	0.0073	0.2113	−0.2185	0.1267	0.0949	0.0252	0.0208	0.2359	0.0164	0.0105	0.0001	0.0403	0.144195
	cg04610450	0.1700	−0.0442	0.0176	0.0171	0.2008	−0.3036	0.1904	0.1214	0.0282	0.0204	0.1780	0.0134	0.0094	0.0003	0.0389	0.117742
PRKCZ	cg02481000	0.1183	−0.0434	0.0213	0.0501	0.2340	−0.3056	0.1538	0.0560	0.0283	0.0194	0.1546	0.0133	0.0099	0.0007	0.0412	0.11632
TLR6	cg14578677	0.1978	−0.0707	0.0256	0.0095	0.2094	−0.2213	0.1301	0.0992	0.0260	0.0207	0.2187	0.0156	0.0116	0.0008	0.0470	0.137144
TNFRSF1B	cg05599723	0.1221	−0.0465	0.0224	0.0462	0.2060	−0.2470	0.1488	0.1075	0.0301	0.0198	0.1387	0.0115	0.0086	0.0003	0.0350	0.100689
ZAP70	cg08859278	0.1844	0.0475	0.0179	0.0126	0.2172	0.3310	0.1844	0.0828	0.0259	0.0204	0.2142	0.0157	0.0093	0.0019	0.0405	0.137839

None of the mediation effects survived Bonferroni correction

**Table 5:** Significant mediating effects of gene methylation on IL-13 outcome

Gene	CpG site	DNA methylation				IL-13 outcome							Mediation effect				
		*R* ^2^	Storm32			*R* ^2^	DNA methylation			Storm32							
			Effect (*a*)	SE	*p* val.		Effect (*b*)	SE	*p* val.	Effect (*c'*)	SE	*p* val.	Effect (a*b)	Boot SE	Boot LLCI	Boot ULCI	Standardized Effect
LCK	cg17078393	0.2561	0.0659	0.0202	0.0027	0.1906	0.1831	0.1123	0.1133	0.0144	0.0146	0.3331	0.0121	0.0087	0.0003	0.0348	0.157233
	cg05350315	0.1949	0.0711	0.026	0.0101	0.1727	0.1235	0.0883	0.1721	0.0177	0.0142	0.223	0.0088	0.0068	0.0001	0.028	0.144773

None of the mediation effects survived Bonferroni correction

#### Adaptive cytokine levels: Th1

##### IFN-γ

As shown in Table 2, 40 of the 47 CpG sites were found to significantly mediate the effect of objective PNMS (Storm32) on IFN-γ cytokine secretion: all of these mediations were negative, that is, the value of a*b is negative, and the confidence interval does not contain 0. These 40 CpGs correspond to 18 genes, whose functions are listed in Additional file 1. After Bonferroni correction, however, six CpGs in six different genes (*PIK3CD*, *PIK3R2*, *NFKBIA*, *TRAF5*, *TNFRSF1B*, and *LTBR*) remained as significant mediators. For three of these CpG sites, the magnitude of objective PNMS exposure was associated with an increase in DNA methylation (i.e., the value of path *a* is positive), which in turn, was associated with a reduction in IFN-γ levels (i.e., the value of path *b* is negative). For the other three CpG sites, the magnitude of objective PNMS exposure was associated with a reduction in DNA methylation (i.e., the value of path *a* is negative) which in turn, was associated with an increase in IFN-γ production (i.e., the value of path *b* is positive). Interestingly, the mediation analyses show that, controlling for DNA methylation, objective PNMS had a positive direct effect (i.e., the values of path *c’* was positive), but a negative mediation effect, on IFN-γ secretion, which explains why the correlation between objective PNMS and IFN-γ was not significant. Together, the combination of Storm32 objective stress and DNA methylation of these six CpGs explained between 17.49 and 24.89 % of the variance in IFN-γ secretion.

##### IL-2

As presented in Table 3, there were nine CpGs, mapping to four genes, which positively mediated the effect of objective PNMS on IL-2 levels. The combination of objective PNMS and DNA methylation explained between 14.58 and 20.09 % of the variance in IL-2 levels. However, none of the CpGs associated with IL-2 outcomes were significant mediators after correcting for multiple testing.

#### Adaptive cytokine levels: Th2

##### IL-4

Table 4 presents the results for IL-4. In total, 12 CpGs, corresponding to 10 genes, positively mediated the effect of objective PNMS on IL-4 levels. Together, objective PNMS and DNA methylation explained 20.35 to 24.57 % of the variance in IL-4. However, none of the CpGs associated with IL-4 outcomes were significant mediators after correcting for multiple testing.

##### IL-13

As shown in Table 5, two CpGs corresponding to *LCK* positively mediated the effect of objective PNMS on IL-13 production. The two models presented explained 17.27 and 19.06 % of the variance in IL-13, respectively. However, none of the CpGs associated with IL-13 outcomes were significant mediators after correcting for multiple testing.

## Discussion

We have shown previously in Project Ice Storm that prenatal maternal stress influences both DNA methylation [23] and cytokine levels [14]. The main challenge in human DNA methylation-association studies is to determine relationships between exposure and DNA methylation and between these changes in DNA methylation and phenotypic outcome. The quasi-random assignment of objective hardship to pregnant women during the 1998 Quebec ice storm offers a unique opportunity to address this issue by combining mediation analyses with the quasi-experimental design. The aim of this study was to determine whether DNA methylation in T cells mediates the effect of objective PNMS exposure on Th1- and Th2- cytokine production in these same children at 13½. Twenty genes from the NF-κB signaling pathway were selected to test for mediation effects. DNA methylation levels of six CpGs corresponding to six genes were significant mediators of the relationship between objective PNMS and IFN-γ levels after correcting for multiple testing. However, no mediation effects on IL-2, IL-4, and IL-13 survived correction. This is the first study to demonstrate that DNA methylation mediates the relationship between PNMS due to a natural disaster and Th1- cytokine production in children.

Similar to our previous report investigating the effect of objective PNMS on both central adiposity and BMI through DNA methylation of genes from established type 1 and 2 diabetes mellitus pathways [24], we also demonstrated here a negative mediating role of DNA methylation of genes from NF-κB pathway on the effect of objective PNMS on IFN-γ; six CpGs from *PIK3CD*, *PIK3R2*, *NFKBIA*, *TRAF5*, *TNFRSF1B*, and *LTBR* survived Bonferroni correction. Interestingly, the highest average negative mediation effect was found in *PIK3CD*, followed by *PIK3R2* (Table 2, path *a*). Both genes belong to the phosphatidylinositol 3-kinase (*PI3K*) gene family [44]. PI3K is a lipid kinase that phosphorylates phosphatidylinositol and similar compounds, generating second messengers important for growth signaling pathways [44, 45]. For instance, evidence of the association between PI3K and IFN-γ has been previously reported in a study showing that the pro-angiogenic effect of IFN-γ on human retinal pigmented epithelial cells depended on the PI3K/mTOR/translational pathway [46]. Furthermore, the regulatory functions of PI3K on IFN-mediated signaling (including IFN-γ which belongs to type II class of interferons) are well-documented (reviewed in [47]).

The immune system is composed of multiple interdependent cellular and molecular components, each one tightly regulated by intrinsic and extrinsic mechanisms. Cytokines play a central regulatory role in the orchestration of the immune response. Thus, any functional changes in cytokine production would have a global impact on the immune response. The overall immune alteration observed in our cohort of 13 year-old participants exposed to maternal stress in utero is a shift in the normal balance of T helper-derived cytokine production, favoring a Th2 pattern as seen in our correlational paper [14]; this shift is seen here in the positive correlation between objective PNMS and the Th2 cytokines (IL-4 and IL-13) and the negative mediation of the effect of objective PNMS on the Th1 cytokine IFN-γ.

Hence, the findings presented here allow us to draw a continuous link that starts with a noxious environmental exposure (PNMS) that alters gene regulation through epigenetic changes (DNA methylation) which in turn induces a pathophysiological alteration (Th1/Th2 cytokine imbalance). Testing the clinical consequences of these changes is beyond the scope of the present work. However, since the presence of a Th2 imbalance has been previously associated with asthma and other allergic disorders (reviewed in [48]), we can hypothesize that PNMS is a factor that increases the risk for the development of these immunological conditions through epigenetic changes in genes responsible for the regulation of the cytokine response.

NF-κB is one of the most important regulators of pro-inflammatory gene expression [49]. The role of NF-κB signaling in chronic inflammatory airway disease has been well documented. An enhanced activation of the NF-κB pathway in asthmatic tissues from animal models and human studies has been reported (reviewed in [50]). As such, the fact that genes we selected from NF-κB signaling pathway had mediating effects on Th1-type cytokine production provides further evidence for the potential contribution of these genes to the susceptibility to asthma. Taken together, our findings may shed some light on the epigenetic mechanisms underlying the association between PNMS and immune-mediated disorders, particularly asthma.

Potential limitations of the present study included the relatively small sample size which restricted statistical power, preventing us from conducting sex-specific analyses. Although this pilot study provides the first evidence that DNA methylation changes on NF-κB pathway-related genes mediate the association between objective PNMS and cytokine outcomes, larger sample sizes are warranted to further validate these effects. Moreover, perhaps due to the small sample size, the mediating effect of many CpGs did not resist the conservative Bonferroni correction for multiple comparisons. Lack of RNA samples due to the low amount of blood collected hindered us from determining the expression levels of NF-κB pathway-related genes, such as *PIK3CD* and *PIK3R2*. Additionally, both the methylation and gene expression levels of cytokine genes such as *IL-2*, *IL-4*, *IL-13*, and *IFN-γ* and their associated pathways need to be further analyzed. Furthermore, the lack of birth tissue from this cohort, such as cord blood, hinders the investigation on whether the methylation alteration was induced early in development and/or was present in pluripotent cells.

The availability of samples taken at birth would have provided an additional level of evidence supporting direct causality, as postnatal events also shape immune function. However, animal studies on prenatal stress and immune development indicate that the changes induced on immunity by PNMS persist well after birth, as they are found at different stages of postnatal development (stages correspondent to human childhood, adolescence and adulthood) [11]. As such, despite the lack of a picture on methylation at birth, our study still predicts a significant proportion of the variability, illustrating how pervasive immune changes caused by PNMS can be. Moreover, this relationship was still significant despite the fact that our participants come from a population with access to adequate health services and high parental education levels, which would have acted as protective (and not risk) factors. Further research with larger samples and access to tissues at birth is warranted. Finally, although we explored the dose-response associations between PNMS and child outcomes, our project lacks data from a control group. However, our study design and previous research has not hindered us from providing solid evidence on the effects of PNMS on multiple physical and psychological outcomes.

This is the first study to demonstrate that DNA methylation mediates the relationship between PNMS due to a natural disaster and adaptive T cell cytokine production in children. Our findings highlight the important role that epigenetic changes—specifically, DNA methylation—have on mediating the effects of PNMS on cytokine production. Our findings provide new data suggesting that DNA methylation could act as an intervening variable between PNMS and immune outcomes. Unlike Project Ice Storm, with which we were able to build a human model with a quasi-randomly assigned and an independent “objective” stressor, other PNMS studies that assess maternal characteristics such as mood during pregnancy are unable to disentangle genetic mechanisms from those of the intrauterine or postnatal environments. Although the reason behind this objective PNMS effect is still unclear, it may be due to the cascade of physiological effects. For example, 40 % of the mothers in the present study reported the fear of being cold during the crisis. As such, we may reasonably hypothesize that maternal exposure to cold temperatures during the ice storm could alter system-wide functioning, including the immune system. Finally, we conducted a mediation analysis using bootstrapping which is a powerful approach because it takes into account the fact that the sampling distribution of the mediated effect is skewed away from 0 allowing it to be applied to small-to-moderate samples [51].

## Conclusions

Six of the negative mediation effects of objective PNMS on IFN-γ, from six NF-κB signaling genes (*PIK3CD*, *PIK3R2*, *NFKBIA*, *TRAF5*, *TNFRSF1B*, and *LTBR*), were significant after Bonferroni correction. The present study provides evidence supporting the mediating role of DNA methylation on the association between objective aspects of PNMS and child immune outcomes, highlighting the importance of fetal programming through epigenetic mechanisms in response to environmental factors.

## Additional file

Additional file 1: Selected 47 CpGs corresponding to 20 genes from NF-κB Signaling. The functions of the selected 20 genes (47 CpGs) involved in the NF-κB signaling pathway. (PDF 175 kb)
