# Supplementary material for: DNA methylation mediates the effect of exposure to prenatal maternal stress on cytokine production in children at age 13½ years: Project Ice Storm
Source: Clin Epigenetics. 2016 May 12;8:54. doi: 10.1186/s13148-016-0219-0 (PMC4866030; doi:10.1186/s13148-016-0219-0)
Supplement: Additional file 1: — Selected 47 CpGs corresponding to 20 genes from NF-κB Signaling. The functions of the selected 20 genes (47 CpGs) involved in the NF-κB signaling pathway. (PDF 175 kb) [file 13148_2016_219_MOESM1_ESM.pdf]

Additional File 1. Selected 47 CpGs corresponding to 20 genes from NF-κB Signaling---

CpG sites	Symbol	Description	Function[a]
cg01636910	BCL10	B-Cell CLL/Lymphoma 10	The protein encoded by this gene contains a caspase recruitment domain (CARD), and has been shown to induce apoptosis and to activate NF-kappaB.
cg11975790	DDR1	Discoidin Domain Receptor Tyrosine Kinase 1	The protein encoded by this gene belongs to a subfamily of tyrosine kinase receptors with homology to Dictyostelium discoideum protein discoidin I in their extracellular domain, and that are activated by various types of collagen.
cg24850711	FLT1	Fms-Related Tyrosine Kinase 1	This gene encodes a member of the vascular endothelial growth factor receptor (VEGFR) family. This protein binds to VEGFR-A, VEGFR-B and placental growth factor and plays an important role in angiogenesis and vasculogenesis.
cg13297560	IGF1R	Insulin-Like Growth Factor 1 Receptor	This receptor binds insulin-like growth factor with a high affinity. It has tyrosine kinase activity. The insulin-like growth factor I receptor plays a critical role in transformation events.
cg25830379			
cg12910851	IL1R2	Interleukin 1 Receptor, Type II	The protein encoded by this gene is a cytokine receptor that belongs to the interleukin 1 receptor family.
cg11683242	LCK	Lymphocyte-Specific Protein Tyrosine Kinase	This gene is a member of the Src family of protein tyrosine kinases (PTKs). The encoded protein is a key signaling molecule in the selection and maturation of developing T-cells.
cg17078393			
cg05350315			
cg14781242			
cg20239639			
cg00501919	LTA	Lymphotoxin Alpha	The encoded protein, a member of the tumor necrosis factor family, is a cytokine produced by lymphocytes.
cg01157951			
cg13815684			
cg14441276			
cg16280132			
cg17709873			
cg26348243			
cg02402436			
cg09621572			
cg09736959			
cg10476003			
cg11586857			
cg14437551			
cg14597739			
cg16219283			
cg17169196			
cg21999229			
cg24216966			
cg23079808	LTBR	Lymphotoxin Beta Receptor	This gene encodes a member of the tumor necrosis factor receptor superfamily. The encoded protein

		(TNFR Superfamily, Member 3)	plays a role in signalling during the development of lymphoid and other organs, lipid metabolism, immune response, and programmed cell death.
cg16826777	MAP3K14	Mitogen-Activated Protein Kinase Kinase Kinase 14	This gene encodes mitogen-activated protein kinase kinase kinase 14, which is a serine/threonine protein-kinase. This kinase binds to TRAF2 and stimulates NF-kappaB activity.
cg03974193	MAP4K4	Mitogen-Activated Protein Kinase Kinase Kinase Kinase 4	The protein encoded by this gene is a member of the serine/threonine protein kinase family. This kinase has been shown to specifically activate MAPK8/JNK.
cg00689225<br>cg16518861	NFKBIA	Nuclear Factor Of Kappa Light Polypeptide Gene Enhancer In B-Cells Inhibitor, Alpha	This gene encodes a member of the NF-kappa-B inhibitor family, which contain multiple ankrin repeat domains. The encoded protein interacts with REL dimers to inhibit NF-kappa-B/REL complexes which are involved in inflammatory responses.
cg01320698<br>cg07499142	PIK3CD	Phosphatidylinositol-4,5-Bisphosphate 3-Kinase, Catalytic Subunit Delta	The protein encoded by this gene is a class I PI3K found primarily in leukocytes. Phosphoinositide 3-kinases (PI3Ks) phosphorylate inositol lipids and are involved in the immune response.
cg04610450<br>cg11953794	PIK3R2	Phosphoinositide-3-Kinase, Regulatory Subunit 2 (Beta)	The protein encoded by this gene is a regulatory component of PI3K.
cg02481000	PRKCZ	Protein Kinase C, Zeta	Protein kinase C (PKC) zeta is a member of the PKC family of serine/threonine kinases which are involved in a variety of cellular processes such as proliferation, differentiation and secretion.
cg04916416	TGFBR2	Transforming Growth Factor, Beta Receptor II	This gene encodes a member of the Ser/Thr protein kinase family and the TGFB receptor subfamily.
cg14578677	TLR6	Toll-Like Receptor 6	The protein encoded by this gene is a member of the Toll-like receptor (TLR) family which plays a fundamental role in pathogen recognition and activation of innate immunity.
cg22014112	TNFAIP3	Tumor Necrosis Factor, Alpha-Induced Protein 3	The protein encoded by this gene is a zinc finger protein and ubiquitin-editing enzyme, and has been shown to inhibit NF-kappa B activation as well as TNF-mediated apoptosis.
cg05599723<br>cg15526535<br>cg22677556	TNFRSF1B	Tumor Necrosis Factor Receptor Superfamily, Member 1B	The protein encoded by this gene is a member of the TNF-receptor superfamily.
cg10721755	TRAF5	TNF Receptor-Associated Factor 5	The scaffold protein encoded by this gene is a member of the tumor necrosis factor receptor-associated factor (TRAF) protein family
cg08859278	ZAP70	Zeta-Chain (TCR) Associated Protein Kinase 70kDa	This gene encodes an enzyme belonging to the protein tyrosine kinase family, and it plays a role in T-cell development and lymphocyte activation.

[a]The public database GeneCards (http://www.genecards.org) was used for the annotation of the genes
